# A mobile health‐facilitated behavioural intervention for community health workers improves exclusive breastfeeding and early infant HIV diagnosis in India: a cluster randomized trial

**DOI:** 10.1002/jia2.25555

**Published:** 2020-07-03

**Authors:** Nishi Suryavanshi, Abhay Kadam, Nikhil Gupte, Asha Hegde, Savita Kanade, Srilatha Sivalenka, V Sampath Kumar, Amita Gupta, Robert C Bollinger, Anita Shankar, Jane McKenzie‐White, Vidya Mave

**Affiliations:** ^1^ Lakshya, Society for Public Health Education and Research Pune India; ^2^ School of Medicine Johns Hopkins University Baltimore MD USA; ^3^ National AIDS Control Organization New Delhi India; ^4^ Division of Global HIV & TB – India Country Office US Centers for Disease Control and Prevention New Delhi India; ^5^ Bloomberg School of Public Health Johns Hopkins University Baltimore MD USA

**Keywords:** PMTCT uptake, HIV, mhealth, outreach workers, behavioural intervention, India

## Abstract

**Introduction:**

India’s national AIDS Control Organization implemented World Health Organization’s option B+ HIV prevention of mother‐to‐child transmission (PMTCT) guidelines in 2013. However, scalable strategies to improve uptake of new PMTCT guidelines to reduce new infection rates are needed. This study assessed impact of Mobile Health‐Facilitated Behavioral Intervention on the uptake of PMTCT services.

**Methods:**

A cluster‐randomized trial of a mobile health (mHealth)‐supported behavioural training intervention targeting outreach workers (ORWs) was conducted in four districts of Maharashtra, India. Clusters (one Integrated Counselling and Testing Center (ICTC, n = 119), all affiliated ORWs (n = 116) and their assigned HIV‐positive pregnant/postpartum clients (n = 1191)) were randomized to standard‐of‐care (SOC) ORW training vs. the COMmunity home Based INDia (COMBIND) intervention – specialized behavioural training plus a tablet‐based mHealth application to support ORW‐patient communication and patient engagement in HIV care. Impact on uptake of maternal antiretroviral therapy at delivery, exclusive breastfeeding at six months, infant nevirapine prophylaxis, and early infant diagnosis at six months was assessed using multi‐level random‐effects logistic regression models.

**Results:**

Of 1191 HIV‐positive pregnant/postpartum women, 884 were eligible for primary outcome assessment; 487 were randomized to COMBIND. Multivariable analyses identified no statistically significant differences in any primary outcome by study arm. COMBIND was associated with higher uptake of exclusive breastfeeding at two months (adjusted Odds Ratio (aOR), 2.10; 95% CI 1.06 to 4.15) and early infant diagnosis at six weeks (aOR, 2.19; 95% CI 1.05 to 3.98) than SOC.

**Conclusions:**

The COMBIND intervention was easily integrated into India’s existing PMTCT programme and improved early uptake of two PMTCT components that require self‐motivated health‐seeking behaviour, thus providing preliminary evidence to support COMBIND as a potentially scalable PMTCT strategy. Further study would identify modifications needed to optimize other PMTCT outcomes.

## 1 INTRODUCTION

World Health Organization (WHO) HIV Prevention of Mother‐to‐Child Transmission (PMTCT) guidelines have been adopted by many high HIV burden countries [1], including India, yet uptake of these recommendations remains largely sub‐optimal [2, 3, 4, 5, 6, 7, 8], Globally, India contributes the third largest HIV burden and ranks 10th in the annual burden of HIV‐positive women and children. Despite implementing one of the world’s largest national PMTCT programmes, only 47% of India’s estimated 35 225 HIV‐positive pregnant women accessed PMTCT services in 2015 [9]. Effective, scalable strategies are needed to improve PMTCT programme implementation.

India’s national PMTCT programme prioritizes four components of the 2010 revised WHO PMTCT guidelines: (1) ART provision among all HIV‐positive pregnant/breastfeeding women; (2) promotion of exclusive breastfeeding (exclusive breastfeeding) for six months; (3) provision of infant nevirapine prophylaxis for six to twelve weeks among HIV‐exposed, breastfed infants and (4) early infant diagnosis among HIV‐exposed infants [10]. To this end, India’s National AIDS Control Organization (NACO) has established more than 30,000 Integrated Counseling and Testing Centers (ICTCs). All ICTCs are attached to an antenatal clinic and provide cost‐free HIV testing/counselling to pregnant women, refer HIV‐positive women to the nearest regional antiretroviral therapy (ART) centre (multiple ICTCs are linked to a single ART centre), and manage follow‐up of HIV‐exposed infants, including nevirapine prophylaxis and early infant diagnosis. During the initial stages, NACO partnered with State AIDS Control Societies (SACS) and local non‐governmental organizations (NGOs) to implement “place‐shifting” of services from health facilities to the home [11] and “task‐shifting” from highly‐trained providers (i.e. doctors and nurses) to community‐based outreach workers (ORWs) [12] trained to help women navigate barriers in accessing services [13, 14]. Identifying additional, scalable strategies would contribute to reducing the 5% incidence rate of HIV infections in India [9].

**Figure 1:**
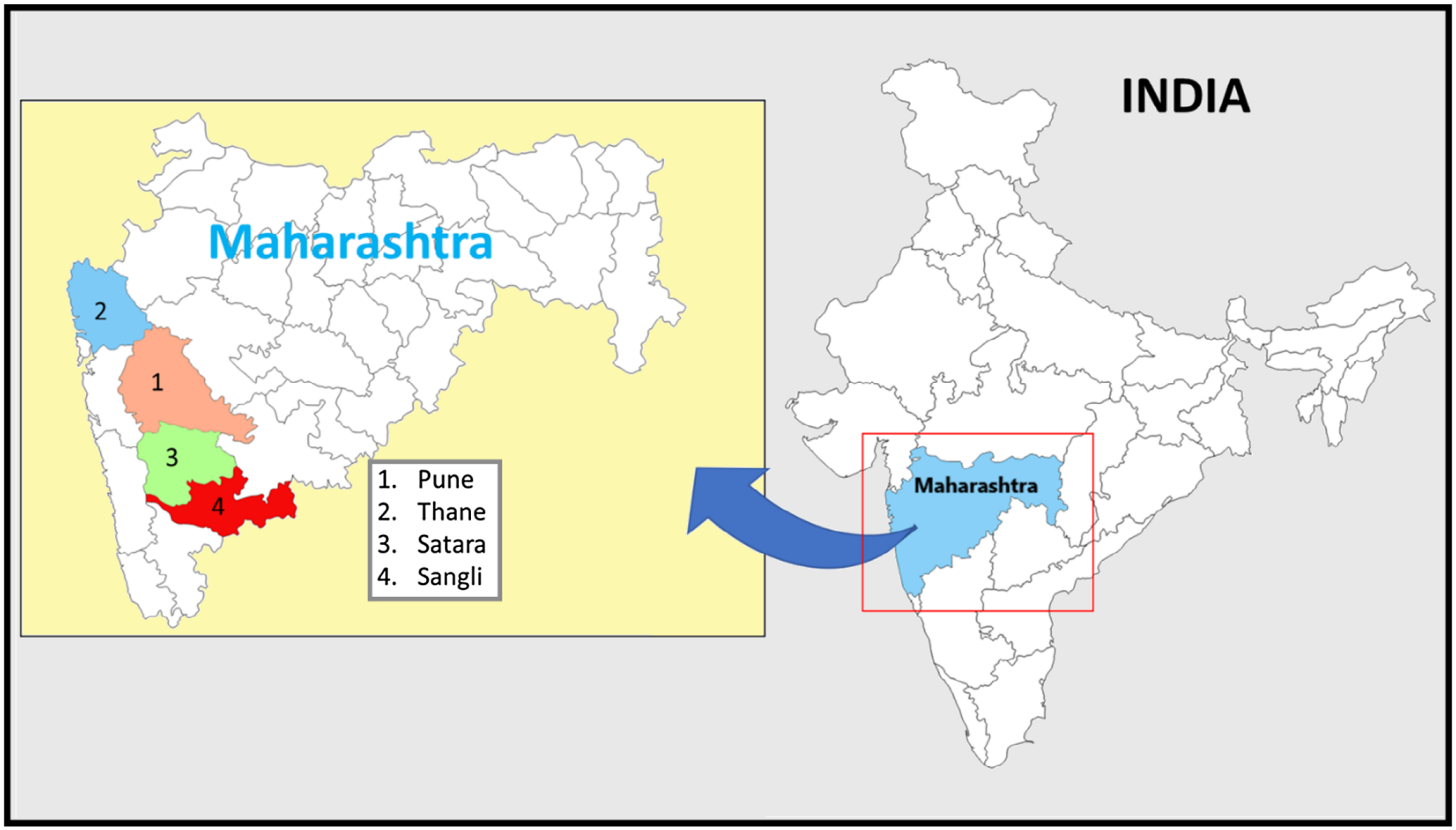
Study districts in Maharashtra state, India. COMBIND study was conducted in four high HIV burden distrcits of Maharashtra state in India; Pune, Thane, Satara and Sangli.

To this end, we collaborated with NACO, the Maharashtra State AIDS Control Society and local NGOs to conduct a cluster‐randomized trial of a mobile health (mHealth)‐facilitated behavioural training intervention, COMmunity Home Based INDia (COMBIND), aiming to increase ORW capacity to assist women in overcoming barriers that limit the uptake of PMTCT services. Since ICTC centres and ORWs (who naturally form a cluster) are responsible for PMTCT services, to eliminate possible contamination of treatment delivery a cluster‐randomized trial design was more appropriate. The study was conducted in Maharashtra state Figure 1 where pregnant women comprise approximately 13,000 of the estimated 747,000 HIV‐positive individuals [15]. We assessed the impact of COMBIND intervention on uptake of the four key components of the newly implemented PMTCT programme in four HIV high‐burden districts of Maharashtra, among HIV‐positive women and their children ( at the individual level).

## 2 METHODS

### 2.1 Study design and population

A cluster‐randomized trial was conducted in four districts (Pune, Thane, Sangli and Satara) that is representative of high HIV‐burden districts and urban and rural populations of Maharashtra state and India from April 2015 to March 2017 Figure 1. A cluster was defined as one ICTC, all enrolled ORWs affiliated with that ICTC (each ORW is assigned to one ICTC), and enrolled HIV‐positive pregnant/postpartum women assigned to their care. ICTCs were randomized to standard‐of‐care (SOC) or COMBIND by study statistician. We used the simultaneous randomization scheme, with randomization stratified by Urban and Rural ICTCs to ensure balance for the assigned treatment strategy, using STATA version 14.2 (copyright 1985 to 2015 StataCorp 4905 Lakeway Drive Figure S1. All HIV‐positive pregnant/postpartum women ≥18 years assigned to enrolled ORWs were eligible, irrespective of pregnancy stage/postpartum status. Four study‐employed Field Coordinators (one per district) consented ORWs affiliated with ICTCs in each district. Enrolled ORWs consented HIV‐positive women assigned to their care.

### 2.2 Study procedures

All study investigators were blinded to the treatment assignment for the entire duration of the study. Study Coordinator received a list of treatment assignments from study statistician and communicated to Field Coordinator inform ICTCs about assigned treatment. Each ICTC centre with consenting ORW formed an eligible cluster to participate in the study. All clusters in the study districts consented to participate in the study. All study data for both the arms were collected following identical procedures by field coordinators and interviewers who were blinded to the randomization assignments of enrolled ORWs and women. Data were collected using a tablet‐based mHealth application (emocha Mobile Health, Baltimore, MD, USA, 2014). Data were captured on SmartForms, encrypted, and wirelessly synced to a secure server based in Lakshya Organization. Field coordinators collected baseline sociodemographic data from ORWs, administered pre‐ and post‐study PMTCT knowledge assessments among ORWs, and made weekly ART centre visits to identify newly registered HIV‐positive women in their district and collected appointment data among enrolled women (missed and upcoming appointments for ART pick up) as well as primary and secondary outcome data Table 1. Recruited interviewers blinded to randomization conducted up to four home visits among enrolled women in both,COMBIND and SOC arms, depending on pregnancy/postpartum status at study entry (baseline visit, within two weeks of delivery, six to eight weeks postpartum, and six months postpartum). Interviewers collected baseline sociodemographic data from HIV‐positive women, administered pre‐ and post‐study PMTCT knowledge assessments among women, and made weekly ICTC visits to collect early infant diagnosis appointment data (upcoming and missed) as well as primary and secondary outcome data Table 1.

**Table 1:** Primary and secondary data description, definitions and data sources for COMmunity home Based INDia Prevention of Mother to Child Transmission (COMBIND‐PMTCT) study (2015 to 2017)

PMTCT component	Description	Definition	Data sources/measurement
Universal antiretroviral treatment (ART) to all HIV‐positive pregnant and breastfeeding women			
Primary outcome measure	Proportion of women on ART at delivery	ART regimen is defined as tenofovir disoproxil fumarate (TDF), lamivudine (3TC) and efavirenz (EFV)	This was transcribed from ART records by Field Coordinators
Exclusive breastfeeding for six months			
Primary outcome measure	Proportion of women Practicing exclusive breastfeeding for six months	Exclusive breastfeeding is defined as infant receiving only breast milk without any additional food or drink	Self‐reports as collected by interviewer Smart form
Secondary outcome measures	Proportion of women Practicing exclusive breastfeeding at two months	exclusive breastfeeding (defined above)	Self‐reports as collected by interviewer smart form
Administration of six‐week extended nevirapine (SWEN) infant prophylaxis			
Primary outcome measure	Proportion of eligible infants who received Nevirapine Prophylaxis	Nevirapine Prophylaxis given in liquid form in the hospital	Documentation from integrated counselling and testing centre (ICTC)/ART centre as collected by interviewer
Breastfeeding care and early infant diagnosis			
Primary outcome measure	Proportion of eligible infants who received early infant diagnosis (DNA PCR) at six months	Eligible infants defined as six months after birth (±1 month)	Laboratory register in ICTC clinic as collected by field coordinator
Secondary outcome measure	Proportion of eligible infants who received early infant diagnosis (DNA PCR) at six weeks, twelve months, and eighteen months Proportion of eligible infants who never missed any early infant diagnosis visit	Eligible infants defined as Infant at week six up to another one month, twelve and eighteen months, ±1 month. All infants who are HIV negative at either week 6, month 6, month 12 or 18, and never missed any of these visits	Laboratory tests of infants from ICTC clinic as collected by field coordinator. Missed visit information as collected by field coordinator from ICTC centre
Maternal mortality Infant mortality	Proportion of women who died Proportion of babies who died	Maternal mortality and infant mortality for the study purpose: Mother who expired before the baby reached 18 months of age. Babies who died before reaching 18 months of age	Documentation from ICTC/ART centre as collected by interviewer

### 2.3 Study arms

Prior to study initiation, ORWs participated in a three‐day, NACO‐mandated HIV training course focused on counselling HIV‐positive women about PMTCT services [14]. ORWs cluster‐randomized to SOC performed all outreach/follow‐up activities required by the Maharashtra State AIDS Control Society programme: (1) register ICTC‐identified HIV‐positive pregnant women with the regional ART centre,(2) provide counselling on ART adherence, exclusive breastfeeding for six months, nevirapine prophylaxis for breastfed infants and early infant diagnosis and (3) conduct monthly home visits during pregnancy and up to 18 months postpartum. In addition to SOC activities, ORWs cluster‐randomized to COMBIND received specialized behavioural training [14] (COMBIND behavioural training component) and a tablet computer loaded with a mHealth application (COMBIND mHealth component) programmed to facilitate: (1) data collection during home visits, (2) ORW‐patient communication during home visits and (3) clinic appointment alerts to patients and ORWs.

### 2.4 COMBIND intervention

#### 2.4.1 Behavioural training component

An intensive one‐week residential training course designed to optimize ORW engagement with HIV‐positive pregnant/postpartum women. Our intervention used an innovative adaptation of a situated information‐motivation‐behavioural skills model of care initiation and maintenance (sIMBCIM) [16] and incorporated following approaches,personal empowerment exercises to increase self‐awareness as well as work‐related motivation and confidence [17], active learning techniques to practice counselling strategies, including motivational interviewing [18, 19], and didactic lectures to fill knowledge gaps (e.g. scientific basis for PMTCT guidelines). In addition, the COMBIND study team developed PMTCT counselling scripts and education videos (via a video production agency contract) in the local language (Marathi and Hindi). Standardized counselling scripts were designed to promote consistent messaging among ORWs while assisting patients in overcoming known barriers (lack of motivation, lack of adequate information on HIV transmission, treatment, adherence, lack of training). Videos depicted HIV‐positive women making decisions concerning: HIV testing during pregnancy; ART registration, ART initiation, side effects of ART and ART adherence; strategies to overcome fear of foetal harm; infant nevirapine prophylaxis; exclusive breastfeeding; early infant diagnosis up to 18 months; importance HIV status disclosure and HIV testing of spouse. Video scripts were authored by NS, AK and SK and validated using national PMTCT guidelines; the video on exclusive breastfeeding was adapted from Healthphone.org [20] and dubbed in Marathi. Lastly, field coordinators monitored ORWs via unannounced field visits and met with ORWs in‐person to provide monthly feedback on the rate of missed patient visits and examine strategies to support improved health‐seeking behaviours.

#### 2.4.2 mHealth component

A mHealth application accessed via a tablet computer provided: an enrolled patient list; a SmartForm to guide home visits/facilitate data collection; access to a library of COMBIND counselling scripts and education videos and appointment notifications (women and infants). All content was in the local language, Marathi. During monthly home visits, ORWs administered the SmartForm, a series of questions gauging patient uptake of PMTCT services. Programmed algorithms facilitated efficient movement between questions based on pregnancy/breastfeeding status and responses to previous questions. Specific responses triggered prompts for the ORW to read one or more counselling scripts and/or show one or more educational videos. For example if a woman’s response indicates that she does not know how to disclose her HIV status, then the application would trigger a script to guide the ORW on counselling about disclosure, or if the woman was breastfeeding, the application would prompt the ORW to show the exclusive breastfeeding video. Lastly, the mHealth application was programmed to send automatic short‐text message service (SMS) alerts to ORW and client cell phones. SMS alerts are triggered by appointment data recorded by field coordinators and interviewers using a SmartForm that is linked to the patient and assigned ORW. SMS scripts were general in nature (e.g. “*This is a reminder that you have an appointment scheduled for [Date and time]” or “This is a message to inform you that you missed your/your baby’s appointment scheduled for [Date and time]”. “Your health is important to us. You are encouraged to come to the clinic within the next week, at any time”*).

### 2.5 Outcomes

Primary study outcomes were as follows: (1) proportion of women on ART at delivery; (2) proportion of infants receiving exclusive breastfeeding at six months; (3) proportion of infants receiving six to twelve weeks of nevirapine [1, 21] and (4) proportion of infants screened for HIV infection at age six months. Secondary outcomes included: exclusive breastfeeding at two months, early infant diagnosis at six weeks, twelve months and eighteen months; missed early infant diagnosis visits and all‐cause maternal and infant mortality. Definitions of outcomes, data sources and measurement are shown in Table 1. All outcomes were measured among HIV‐positive women and their children (at individual level).

### 2.6 Statistical analysis

Primary outcomes were powered at 80% to detect an absolute difference of at least 12% among study arms at a 5% level of significance with a coefficient of variation between 0.25 and 0.50, yielding a sample size of 900 HIV‐positive pregnant/postpartum women and their children [13]. Methods described in “Statistical analysis and optimal design for cluster randomized trails [22], were used and the programming was done in statistical software R for sample size and power calculations. Continuous variables were summarized using medians and interquartile range (IQR) and compared using Wilcoxon rank‐sum test,discrete variables were summarized using frequencies and proportions and compared using Fisher’s exact test. Outcomes were measured on individuals with a multi‐level framework. The levels were District ‐>ICTC ‐> ORW ‐> Individual patient. The Effect if COMBIND arm was assessed using a multi‐level random effect logistic regression with random effects for district, ICTC and ORW and fixed effect for treatment arm to derive unadjusted and adjusted odds ratio (OR) and 95% CIs for primary and secondary outcomes. Variables selected for the multivariable analysis were the ones that were significant in the univariable analysis and those were known to be associated with the outcomes. To assess if a variable was eligible for multivariable analysis, we used a *p*‐value cut‐off of 10% and to assess if the variable is independently associated with outcome a level of significance of 5% was used. Data were analysed using STATA version 14.2 (copyright 1985 to 2015 StataCorp 4905 Lakeway Drive, TX, USA).

### 2.7 Ethics statement

The study protocol was reviewed, approved and received ethical clearance from the ethics committee (FWA00016806) of Lakshya Society for Public Health Education (Lakshya), the Institutional Review Board at the Johns Hopkins University School of Medicine, the NACO research committee, and the science office of US Centers for Disease Control and Prevention.

## 3 RESULTS

### 3.1 Baseline characteristics

**Figure 2:**
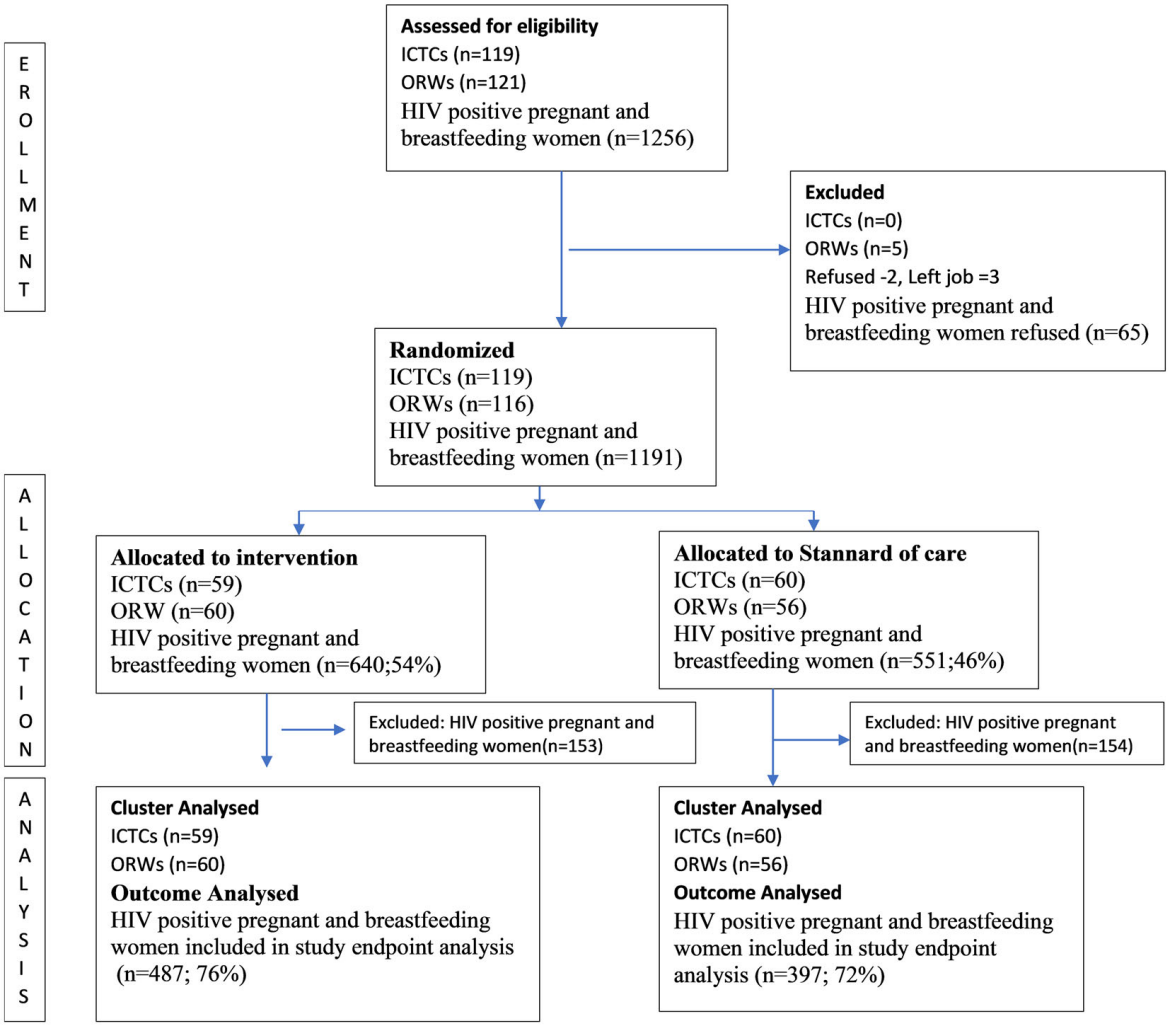
Flow of clusters and participants in COMBIND study. The study clusters were ICTc centres (119), ORWs (121) and HIV‐positive pregnant and postpartum women (1256). Randomization included 119 ICTCS, 116 ORWs and 1191 enrolled HIV‐positive women. Fifty‐nine ICTCs, 60 ORWs and 640 HIV‐positive women were randomized in intervention arm. Notably, 153 HIV‐positive women from intervention arm and 154 from SOC arm had completed six months of breastfeeding at the time of study enrolment and could only be included in secondary outcome analysis. The remaining 884 pregnant/postpartum women were eligible for at least one primary outcome assessment; of these, 487 and 397 were in the Intervention and SOC arms respectively.

A total of 119 ICTCs were cluster‐randomized Figure S1. Of 116 enrolled ORWs, 60 (52%) were randomized to COMBIND. Baseline characteristics were comparable by study arm Table S1,overall, the median age was 37 (IQR, 35 to 42) years, and 86 (74%) were from the Pune and Thane districts. Among 1191 enrolled HIV‐positive women, 553 were pregnant and 638 were postpartum at study entry. Notably, 307 of postpartum women had completed six months of breastfeeding and could only be included in secondary outcome analysis Table S2. The remaining 884 pregnant/postpartum women were eligible for at least one primary outcome assessment,of these, 487 and 397 were in the COMBIND and SOC arms respectively Figure 2. Baseline maternal characteristics were comparable by study arm Table 2. Overall, the median age was 25 years (IQR, 22 to 29), the majority (77%) were from the Pune and Thane districts, the median duration of HIV diagnosis was 1.21 years (IQR, 0.26 to 3.51), and 1035 (99.8%) were on ART with a median CD4 count of 420 cells/mL (IQR, 248 to 587). Overall the median (IQR) of the cluster size was 8 (5 to 15).

**Table 2:** Characteristics of HIV‐positive pregnant and breastfeeding women and their children by study arms randomized in COMmunity home Based INDia Prevention of Mother to Child Transmission (COMBIND‐PMTCT) study in Maharashtra, India

Characteristics	Overall (n = 1191)	COMBIND (n = 640)	SOC (n = 551)	*p*‐value
District				
Pune	422 (35%)	227 (54%)	195 (46%)	0.003
Sangli	134 (11%)	82 (61%)	52 (39%)	
Satara	137 (12%)	88 (64%)	49 (36%)	
Thane	498 (42%)	243 (49%)	255 (51%)	
Age, median (IQR)	25 (22 to 29)	25 (22 to 29)	25 (22 to 29)	0.14
Stage of study entry				
Pregnant	552 (46%)	311 (56%)	241 (44%)	
<6 weeks. PP	105 (9%)	60 (57%)	45 (43%)	0.19
6 weeks. to 6 months PP	227 (19%)	116 (51%)	111 (49%)	
>6 months. PP	307 (26%)	153 (50%)	154 (50%)	
HIV‐positive children				
No	783 (89%)	407 (52%)	376 (48%)	0.50
Yes	87 (10%)	50 (57%)	37 (43%)	
Refused	13 (1%)	9 (69%)	4 (31%)	
Registered for ART	1037 (99.8%)	569 (55%)	468 (45%)	
Not registered for ART	2 (0.2%)	1 (50%)	1 (50%)	>0.95
ART initiated				
No	2 (0.2%)	2 (100%)	0	
Yes	1035 (99.8%)	567 (55%)	468 (45%)	
Baseline CD4, Median (IQR)	420 (248 to 587)	402 (247 to 576)	432 (249 to 592)	0.31
Recent median CD4, (IQR)	558 (365 to 742)	540 (366 to 742)	572 (364 to 749)	0.67
Median years since HIV, (IQR)	1.21 (0.26 to 3.51)	1.26 (0.27 to 4.02)	1.19 (0.24 to 3.28)	0.26
Median years since ART, (IQR)	0.77 (0.16 to 1.67)	0.77 (0.16 to 1.90)	0.77 (0.16 to 1.59)	0.58

The overall column percentage is the distribution of the variable in our data. However, the row percentage is the distribution of a particular category of a variable across the arms. The “overall” is column percentage, and the percentage in the “COMBIND” and “SOC” are row percentage. ART, anti‐retroviral therapy; ORW, outreach workers; PHC, Primary Health Center; PMTCT, prevention of mother to child transmission; PP, Post‐partum; SOC, standard of care.

### 3.2 Effect of COMBIND intervention on uptake of PMTCT programme

**Figure 3:**
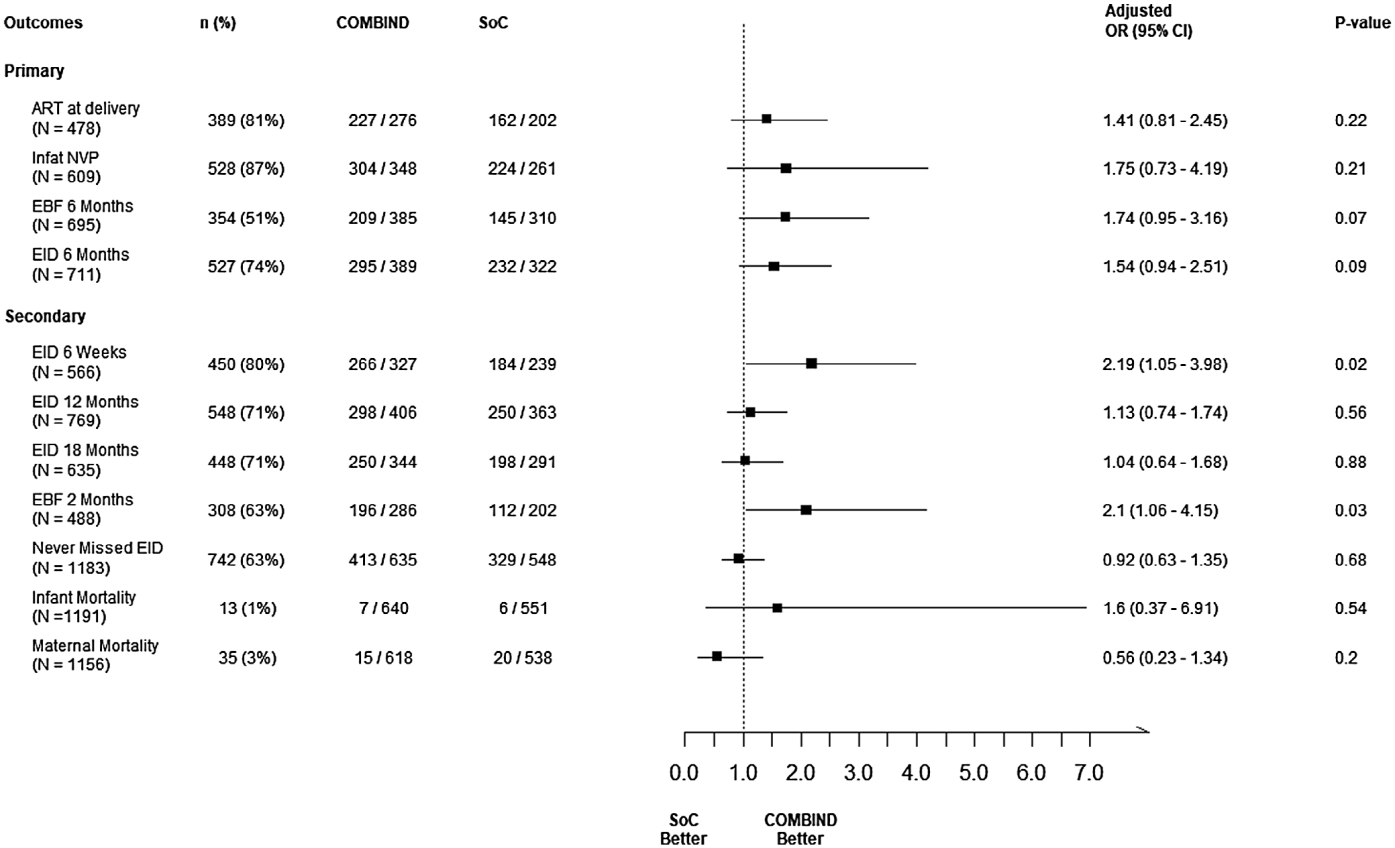
Effect of COMmunity Home Based INDia (COMBIND) intervention on the uptake of national PMTCT services in Maharashtra, India. This figure depicts that COMBIND intervention showed no statistically significant differences between study arms in any primary outcome after adjusting for maternal and ORW characteristics. However, Compared to SOC, the COMBIND arm had twofold higher uptake of both exclusive breastfeeding at two months (adjusted odds ratio (aOR), 2.10; 95% CI, 1.06 to 4.15) and early infant diagnosis at six weeks (aOR, 2.19; 95% CI, 1.05 to 3.98). No significant differences in maternal or infant death by study arm were observed.

Of the 884 women included in the primary outcome analysis, 759 (86%) had the desired outcome for at least one endpoint. In multivariable analysis, we have estimated the intracluster correlation (ICC) for the primary outcomes. The for ART at delivery is 0.10, Infant NVP is 0.003, Exclusive BF for six months is 0.07 and EID at six weeks is 0.01. There were no statistically significant differences between study arms in any primary outcome after adjusting for maternal (age, occupation, education, family type and HIV status of the previous child) and ORW (age, education and HIV status) characteristics Figure 3, Table S2. Compared to SOC, the COMBIND arm had twofold higher uptake of both exclusive breastfeeding at two months (adjusted odds ratio (aOR), 2.10; 95% CI, 1.06 to 4.15) and early infant diagnosis at 6 weeks (aOR, 2.19; 95% CI, 1.05 to 3.98). Overall, there were 13 maternal and 35 infant all‐cause deaths with no significant differences in maternal (1.0% vs. 1.1%; *p *> 0.95) or infant (2.3% vs. 3.6%; *p* = 0.54) deaths by study arm. Among 864 available results, the proportion of HIV‐positive infants at age six weeks and six months was 2% in both arms. Lost‐to‐follow‐up (due to migration, refusal) was 4% across both arms. The proportion of infants with no missed appointments was comparable in COMBIND and SOC (65% vs. 60%; *p* = 0.09).

## 4 DISCUSSION

Recent systematic reviews failed to identify evidence to support specific strategies to improve delivery of the WHO‐recommended option B+ PMTCT programme in resource‐limited, high HIV burden countries. [23, 24], Our cluster‐randomized pragmatic study of a mHealth‐facilitated ORW behavioural training intervention (COMBIND) conducted in four high HIV burden districts identified the overall robust implementation of option B+ guidelines in India’s national programme, twofold higher early uptake of key secondary outcomes – exclusive breastfeeding and early infant diagnosis in the COMBIND arm versus SOC, following COMBIND intervention. Although COMBIND had no statistically significant impact on any primary outcomes including uptake of maternal ART at delivery or infant nevirapine prophylaxis, our unique intervention shows promise as a scalable strategy. Future studies should be directed at identifying ways to enhance impact in these outcome areas and to achieve sustained impact on uptake of exclusive breastfeeding and early infant diagnosis at six months.

With more than 99% of HIV‐positive women on ART at study entry, our study indicates >200% higher PMTCT uptake among pregnant and breastfeeding women than previously reported for India by UNAIDS in 2018 (60%) [25]. Notably, qualitative studies have shown that the desire to protect children from HIV improves ART adherence during pregnancy and the immediate postpartum period [7] provided the anti‐HIV supply chain remains uninterrupted. However, despite equal access to ART and nevirapine prophylaxis, overall uptake of maternal ART at delivery and infant nevirapine prophylaxis was suboptimal at 83% and 87%, respectively, and did not differ by study arm.

In contrast, the COMBIND intervention was associated with significantly higher early uptake of exclusive breastfeeding (at two months) and early infant diagnosis (at six weeks) than SOC. Early uptake of early infant diagnosis is critical, as early ART initiation among HIV‐positive infants may lead to a functional cure [26, 27]. Similarly, compliance with exclusive breastfeeding is important during the initial months of life, as infants that are mixed fed or not exclusively breastfed have a higher risk of mortality and morbidity [28, 29]. It remains unclear why the impact of COMBIND did not extend to exclusive breastfeeding and early infant diagnosis at six months in multivariable analysis. However, postpartum adherence to PMTCT programmes is known to decrease over time [7, 25]. A modification to the proposed COMBIND strategy may be needed to achieve a sustained effect.

Our central hypothesis was that COMBIND would result in improved PMTCT implementation in each of the four key components of India’s PMTCT programme. Why did COMBIND improve uptake of exclusive breastfeeding and early infant diagnosis, but have no effect on ART at delivery or infant nevirapine prophylaxis? One possible explanation is that, unlike the other two PMTCT components, exclusive breastfeeding and early infant diagnosis require personal‐level motivation [30, 31]. The COMBIND strategies that were designed to actuate behavioural change among ORWs, including the mHealth tools (i.e. SmartForm‐guided home visits plus COMBIND counselling/education tools) and behavioural training strategies (i.e. personal empowerment and motivational interviewing techniques), helped ORWs to improve relational skills and may have cultivated positive attitudes among HIV‐positive women towards acceptance of both exclusive breastfeeding and early infant diagnosis in the short term (qualitative analyses data under peer review conducted a process evaluation to document the impact of COMBIND on the ability of ORWs and women to navigate barriers) [18, 19].

Previous studies have evaluated various PMTCT interventions aiming to improve postpartum retention, optimize the transition from PMTCT to general ART programmes, and improve retention in general ART programmes. [23, 24], While the focus of these studies was to assess the effectiveness of interventions at patient, provider and system levels, these studies were limited by low‐quality evidence, small effect size and showed mixed results of uncertain clinical significance [24]. The comprehensive approach of our study is unique and targeted both provider (ORW) and patient (HIV‐positive women). Uptake of all four key components of India’s PMTCT programme were targeted, and the study population included the entire range of HIV‐positive women who participate in the existing national PMTCT programme (i.e. any pregnancy stage through the postpartum period). In addition, previous mHealth interventions have used SMS messages or phone calls to improve retention of mother and child in care with limited impact [32, 33]. Our study, however, used the mHealth application as a multipurpose tool to optimize: programme‐level data extraction,ORW home visits for tailored education and counselling of HIV‐positive pregnant/breastfeeding women [34] and retention of women/infants in PMTCT care via SMS appointment alerts to patients/ORWs. Importantly, our experience with study implementation in Maharashtra demonstrates that the COMBIND‐PMTCT intervention can be easily integrated into an existing PMTCT outreach programme and scaled across multiple high HIV burden regions of India and beyond.

Our study has several potential limitations. ORWs in the SOC arm may have increased the quality and consistency of their PMTCT services due to the Hawthorne effect [35], which may explain why improved uptake of PMTCT services in COMBIND did not reach statistical significance. The cost‐effectiveness of the interventions was not a part of this study, however, we plan to conduct this analysis in the near future. Finally, the sample size for individual primary and secondary outcomes was lower than originally planned, thus some of the analyses may be underpowered.

## 5 CONCLUSIONS

In conclusion, while evidence for implementation of option B+ programme is robust, there is a critical need to identify strategies for optimal implementation of national PMTCT programmes. Overall, the COMBIND intervention had a significant impact on two key PMTCT outcomes in the short term (approximately two‐months), but no impact on the remaining two outcomes. Using the locally designed mHealth platform and simple‐to‐use, low‐cost tablets to support behavioural training among community‐based ORWs is a promising and potentially scalable strategy but will require modification to be more effective for all key PMTCT outcomes. Future studies evaluating the COMBIND programme would provide insight on the modifications needed before implementation in other contexts.

## COMPETING INTEREST

Under a licensing agreement between emocha Mobile Health, Inc and the Johns Hopkins University, R. Bollinger and J. McKenzie‐White are entitled to royalties on an invention described in this article. R. Bollinger also is a Board of Directors member of emocha Mobile Health, Inc. This arrangement has been reviewed and approved by the Johns Hopkins University in accordance with its conflict of interest policies.

## AUTHORS' CONTRIBUTIONS

NS, VM, NG, AG, RCB, AS, SS and SK conceived the study. NS, VM, JMW RCB, AK, SK and AS prepared smart data collection forms. NS, AK and JMW programmed the forms in eMOCHA software. NS, VM, NG, AK, SK and AH implemented the study and performed data analyses and data interpretation. NS and VM drafted the initial manuscript. RCB, AG, JMW AS, SK, SS and SK critically reviewed the manuscript and provided inputs. All authors approved the manuscript.

## Supporting information


**Figure S1.** Cluster randomization by ICTCs in Maharashtra at four study districts (Pune, Thane, Satara and Sangli).

**Table S1.** Characteristics of outreach workers (ORWs) by study arms randomized in COMmunity home Based INDia Prevention of Mother to Child Transmission (COMBIND‐PMTCT) study in Maharashtra, India

**Table S2.** Effect of COMmunity Home Based INDia (COMBIND) intervention on the uptake of national PMTCT services in Maharashtra, India (Pune, Thane, Satara and Sangli) from April 2015 to March 2017

## Data Availability

All the data required for this paper are presented in the manuscript. The datasets generated during and/or analysed during the COMBIND study are not publicly available due to confidentiality, as they deal with HIV status and other sensitive information related to the participants. However, they may be available from the corresponding author on a reasonable request.
