# Supplementary material for: A mobile health‐facilitated behavioural intervention for community health workers improves exclusive breastfeeding and early infant HIV diagnosis in India: a cluster randomized trial
Source: J Int AIDS Soc. 2020 Jul 3;23(7):e25555. doi: 10.1002/jia2.25555 (PMC7332965; doi:10.1002/jia2.25555)
Supplement: Supplementary file 1 — Figure S1. Cluster randomization by ICTCs in Maharashtra at four study districts (Pune, Thane, Satara and Sangli). [file JIA2-23-e25555-s001.docx]

**Supplementary Figure 1. Cluster Randomization by ICTCs in Maharashtra at four study districts (Pune, Thane, Satara and Sangli).**


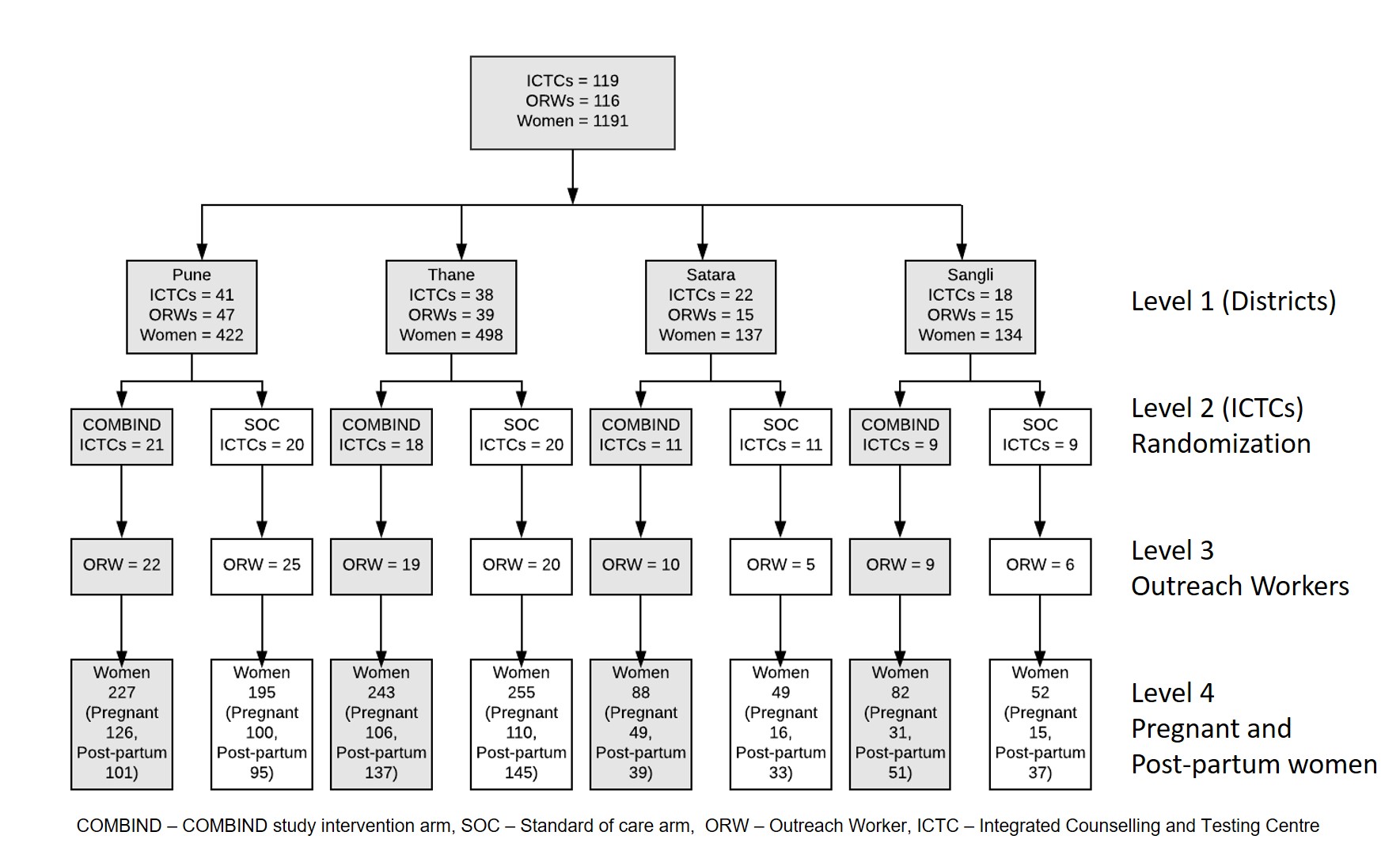


**Level 1**: Level one shows four high HIV burden districts, Pune, Thane, Sangli and Satara of Maharashtra State with number of Integrated counselling and testing centers (ICTCs), Outreach workers (ORWs), and number of pregnant and breastfeeding HIV- infected women who participated in the study.

**Level 2:** Integrated counselling and testing centers (ICTCs) at each study districts and their assigned outreach workers were randomized to standard of care (Arm 1) Vs COMBIND intervention (Arm 2).

**Level 3:** Outreach workers belonging to ICTCs randomized to COMBIND arm were given mHealth and behavioral intervention. Level 3 shows number of ORWs in each study arm at four study districts.

**Level 4:** Study Outcome were measured on HIV- infected pregnant/ breastfeeding women
