# Supplementary material for: A mobile health‐facilitated behavioural intervention for community health workers improves exclusive breastfeeding and early infant HIV diagnosis in India: a cluster randomized trial
Source: J Int AIDS Soc. 2020 Jul 3;23(7):e25555. doi: 10.1002/jia2.25555 (PMC7332965; doi:10.1002/jia2.25555)
Supplement: Supplementary file 2 — Table S1. Characteristics of outreach workers (ORWs) by study arms randomized in COMmunity home Based INDia Prevention of Mother to Child Transmission (COMBIND‐PMTCT) study in Maharashtra, India [file JIA2-23-e25555-s002.docx]

| **Supplementary Table 1. Characteristics of outreach workers (ORWs) by study arms randomized in COMmunity home Based INDia Prevention of Mother to Child Transmission (COMBIND-PMTCT) study in Maharashtra, India.** | | | | |
| --- | --- | --- | --- | --- |
| **Characteristics** | **Overall**  **(n = 116)** | **COMBIND**  **(n = 60)** | **SOC**  **(n = 56)** | **p-value** |
| District  Pune  Sangli  Satara  Thane | 47 (40%)  15 (13%)  15 (13%)  39 (34%) | 22 (47%)  9 (60%)  10 (67%)  19 (49%) | 25 (53%)  6 (40%)  5 (33%)  20 (51%) | 0.55 |
| Age, Median (IQR) | 37 (32 – 40) | 37 (32 – 39) | 38 (32 – 41) | 0.39 |
| Marital Status  Unmarried  Married  Divorced/Widowed/Deserted | 4 (3%)  67 (58%)  45 (39%) | 2 (50%)  30 (45%)  28 (56%) | 2 (50%)  37 (55%)  17(44%) | 0.12 |
| Years of Education  Secondary  Higher  College | 68 (59%)  28 (24%)  20 (17%) | 33 (49%)  13 (46%)  14 (70%) | 35 (51%)  15 (54%)  6 (30%) | 0.20 |
| HIV  Negative  Infected | 65 (63%)  38 (37%) | 33 (51%)  22 (58%) | 32 (49%)  16 (42%) | 0.54 |
| On ART  No  Yes | 3 (8%)  35 (92%) | 1 (33%)  21 (60%) | 2 (67%)  14 (40%) | 0.56 |
| Footnote: PMTCT=Prevention of Mother To Child Transmission, SOC= Standard of care, ART=Anti-retroviral Therapy, ORW= Outreach workers  The overall column percentage is the distribution of the variable in our data. However, the row percentage is the distribution of a particular category of a variable across the arms. The “overall” is column percentage, and the percentage in the “COMBIND” and “SOC” are row percentage | | | | |
