# Supplementary material for: A mobile health‐facilitated behavioural intervention for community health workers improves exclusive breastfeeding and early infant HIV diagnosis in India: a cluster randomized trial
Source: J Int AIDS Soc. 2020 Jul 3;23(7):e25555. doi: 10.1002/jia2.25555 (PMC7332965; doi:10.1002/jia2.25555)
Supplement: Supplementary file 3 — Table S2. Effect of COMmunity Home Based INDia (COMBIND) intervention on the uptake of national PMTCT services in Maharashtra, India (Pune, Thane, Satara and Sangli) from April 2015 to March 2017 [file JIA2-23-e25555-s003.docx]

| **Supplementary Table 2.** **Effect of COMmunity Home Based INDia (COMBIND) intervention on the uptake of national PMTCT services in Maharashtra, India (Pune, Thane, Satara and Sangli) from April 2015 to March 2017.** | | | | |
| --- | --- | --- | --- | --- |
| **Study Endpoints**  **(Eligible participants)** | **COMBIND arm**  **Achieved / Eligible**  **N (%)** | **SOC arm**  **Achieved/Eligible**  **N (%)** | **Unadjusted Odds Ratio (95% CI)** | **Adjusted* Odds Ratio (95% CI)** |
| **Primary endpoints** | | | | |
| Antiretroviral treatment at delivery (n=478) | 227/276 (82) | 162/202 (80) | 1.21 (0.71 – 2.04) | 1.41 (0.81 – 2.45) |
| Nevirapine Prophylaxis for 6 weeks (n=609) | 304 /348 (87) | 224 /261 (86) | 1.15 (0.72 – 1.85) | 1.75 (0.73 – 4.19) |
| Exclusive Breast feeding 6 Months (n=695) | 209 /385 (54) | 145 /310 (47) | 1.82 (1.09 – 3.04) | 1.74 (0.95 – 3.16) |
| Early Infant Diagnosis at 6 Months. (n =711) | 295/389 (76) | 232/322 (72) | 1.22 (0.81 – 1.83) | 1.54 (0.94 – 2.51) |
| **Secondary endpoints** | | | | |
| **Early Infant Diagnosis (EARLY INFANT DIAGNOSIS) at 6 Weeks (n=566)** | **266/327 (81)** | **184/239 (77)** | **1.34 (0.87 – 2.05)** | **2.19 (1.05 – 3.98)** |
| Infant HIV testing at 12 Months (n=769) | 298/406 (73) | 250/363 (69) | 1.24 (0.87 – 1.77) | 1.13 (0.74 – 1.74) |
| Infant HIV testing at 18 Months (n=635) | 250/344 (73) | 198/291 (68) | 1.23 (0.80 – 1.89) | 1.04 (0.64 – 1.68) |
| **Excusive breast feeding at 2 months (n= 488)** | **196/286 (69)** | **112/202 (55)** | **2.05 (1.23 – 3.41)** | **2.10 (1.06 – 4.15)** |
| Not missed any early infant diagnosis visit months of age (n=1183) | 413/635 (65) | 329/548 (60) | 0.84 (0.60 – 1.17) | 0.92 (0.63 – 1.35) |
| Infant death (n= 1156) | 15/618 (2) | 20/538 (4) | 0.68 (0.35 – 1.33) | 0.56 (0.23 – 1.34) |
| Maternal death (n =1191) | 7/640 (1) | 6/551 (1) | 1.06 (0.36 – 3.16) | 1.60 (0.37 – 6.91) |
| SOC=standard of care  * ART at delivery was adjusted for, age, occupation, education, family type, & ORW’s age, years of education and HIV status of ORWs.  Infant Nevirapine was adjusted for, age, occupation, education, family type, having HIV- infected children & ORW’s age, years of education, and HIV status.  *Exclusive breastfeeding at two and six months was adjusted for, age, occupation, education, family type, having HIV- infected children & Outreach worker’s age, years of education, and HIV status.  *early infant diagnosis analysis was adjusted for age, occupation, education, family type, having HIV- infected children & ORW’s age, years of education, and HIV status. | | | | |
|  | | | | |
